# Longitudinal validity of PET‐based staging of regional amyloid deposition

**DOI:** 10.1002/hbm.25121

**Published:** 2020-07-10

**Authors:** Irina Jelistratova, Stefan J. Teipel, Michel J. Grothe

**Affiliations:** ^1^ German Center for Neurodegenerative Diseases (DZNE) Rostock Germany; ^2^ Department of Psychosomatic Medicine University of Rostock Rostock Germany; ^3^ Unidad de Trastornos del Movimiento, Servicio de Neurología y Neurofisiología Clínica, Instituto de Biomedicina de Sevilla Hospital Universitario Virgen del Rocío/CSIC/Universidad de Sevilla Seville Spain

**Keywords:** Alzheimer's disease, amyloid‐PET, in‐vivo amyloid staging, longitudinal progression

## Abstract

Positron emission tomography (PET)‐based staging of regional amyloid deposition has recently emerged as a promising tool for sensitive detection and stratification of pathology progression in Alzheimer's Disease (AD). Here we present an updated methodological framework for PET‐based amyloid staging using region–specific amyloid‐positivity thresholds and assess its longitudinal validity using serial PET acquisitions. We defined region‐specific thresholds of amyloid‐positivity based on Florbetapir‐PET data of 13 young healthy individuals (age ≤ 45y), applied these thresholds to Florbetapir‐PET data of 179 cognitively normal older individuals to estimate a regional amyloid staging model, and tested this model in a larger sample of patients with mild cognitive impairment (*N* = 403) and AD dementia (*N* = 85). 2‐year follow‐up Florbetapir‐PET scans from a subset of this sample (*N* = 436) were used to assess the longitudinal validity of the cross‐sectional model based on individual stage transitions and data‐driven longitudinal trajectory modeling. Results show a remarkable congruence between cross‐sectionally estimated and longitudinally modeled trajectories of amyloid accumulation, beginning in anterior temporal areas, followed by frontal and medial parietal areas, the remaining associative neocortex, and finally primary sensory‐motor areas and subcortical regions. Over 98% of individual amyloid deposition profiles and longitudinal stage transitions adhered to this staging scheme of regional pathology progression, which was further supported by corresponding changes in cerebrospinal fluid biomarkers. In conclusion, we provide a methodological refinement and longitudinal validation of PET‐based staging of regional amyloid accumulation, which may help improving early detection and in‐vivo stratification of pathologic disease progression in AD.

## Graphical abstract

Positron emission tomography (PET)‐based staging of regional amyloid deposition has recently emerged as a promising tool for sensitive detection and stratification of pathology progression in Alzheimer's disease. Here we present an updated methodological framework for PET‐based amyloid staging using region–specific amyloid‐positivity thresholds, and use serial PET acquisitions to provide evidence for the longitudinal validity of the estimated amyloid progression sequence underlying the regional staging scheme.

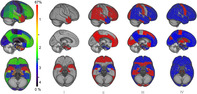

## 1 INTRODUCTION

One of the most important and earliest histopathological hallmarks of Alzheimer's disease (AD) are senile plaques mainly consisting of amyloid‐β (Aβ) protein. Assessment of amyloid pathology extent based on autopsy studies suggests downward amyloid pathology spread from the associative neocortex over limbic and primary sensory‐motor areas to subcortical structures (Braak & Braak, 1991; Braak, Thal, Ghebremedhin, & Del Tredici, 2011; Thal, Rüb, Orantes, & Braak, 2002). Numerous studies have shown that early phases of amyloid accumulation can be observed in cognitively unimpaired older individuals decades before the onset of dementia (Braak et al., 2011; Jack et al., 2013; Sperling, Mormino, & Johnson, 2014; Thal et al., 2002).

Amyloid imaging with positron emission tomography (PET) has become an important diagnostic tool for AD (Villemagne et al., 2011), displaying high sensitivity and specificity by comparison with neuropathological findings (Clark et al., 2012; Sabri, Seibyl, Rowe, & Barthel, 2015; Villeneuve et al., 2015). Recent amyloid PET studies have attempted to characterize and stage the regional amyloid pathology spread in‐vivo, either using an a priori distinction between early neocortical and later subcortical amyloid deposition (Cho et al., 2018; Hanseeuw et al., 2018; Thal et al., 2018) or more comprehensive data‐driven models including regional deposition differences within both cortical and subcortical areas (Cho et al., 2016; Grothe et al., 2017; Sakr et al., 2019). In our previous work (Grothe et al., 2017) we have developed an in‐vivo staging model that adopts a commonly used analytic approach for determining regional staging schemes in neuropathological studies (Braak & Braak, 1991; Josephs et al., 2016; Thal et al., 2002) and is based on the frequency of regionally measured uptake positivity in cognitively normal older participants. The hierarchical stage model showed a progression pattern evolving from temporobasal and frontomedial areas (I), over the remaining associative neocortex (II), to primary sensory‐motor cortex and the medial temporal lobe (III), and finally the striatum (IV). Across individuals the amyloid deposition patterns were highly consistent with the predicted hierarchy across these brain regions, allowing to classify almost all individual profiles (>98%) into one of four progressive amyloid stages. Furthermore, in a follow‐up study we were able to show the validity of this model in an independent cohort consisting of people with subjective memory complaints enrolled in the French INSIGHT‐pre‐AD study (Sakr et al., 2019). The first stages of amyloid accumulation identified by the in‐vivo staging scheme were already paralleled by concomitant decreases in CSF‐Aβ values, but in both cohorts were largely classified as amyloid‐negative by commonly used mean global cortical radiotracer uptake measurement. Moreover, while AD dementia patients unanimously exhibited advanced in‐vivo amyloid stages (III/IV), amyloid burden among nondemented individuals varied across the full spectrum of amyloid stages and correlated with decreased memory scores in these individuals.

Together, the proposed method of in‐vivo amyloid staging shows promising results for early detection of amyloid accumulation and a stratification of the predementia phase of AD based on an individual's extent of amyloid pathology. However, in analogy to neuropathologic staging schemes, the model describes cross‐sectional data, and thus it is still unclear whether this frequency‐based approach reflects actual longitudinal progression patterns. Moreover, it is important to keep in mind that the staging approach also depends on a range of methodological settings that may affect the final staging outcome. In particular, our staging approach as originally devised employed a constant threshold for defining regional amyloid‐positivity, which may not accurately account for differing noise levels across different brain regions. Thus, amyloid PET studies have shown considerable regional signal variations even in young adults (Joshi et al., 2012) who are highly unlikely to exhibit any cerebral amyloid deposition (Braak et al., 2011; Kok et al., 2009), indicating region‐specific signal confounds.

In the present study we updated our original in‐vivo amyloid staging method using a technically more appropriate approach for detecting regional amyloid‐positivity based on region‐specific signal thresholds defined in a young control population, and then further assessed the longitudinal validity of the cross‐sectionally estimated staging model by studying individual amyloid progression patterns in serial PET assessments.

## 2 MATERIALS AND METHODS

### 2.1 Participants

As in our previous staging study (Grothe et al., 2017), we included baseline Florbetapir‐PET scans of 667 participants from the Alzheimer's Disease Neuroimaging Initiative (ADNI) database (http://adni.loni.usc.edu/). The sample comprised 179 cognitively normal older controls (CN), 403 participants with mild cognitive impairment (MCI), and 85 Alzheimer's disease dementia patients (AD). Clinical assessment criteria for each of the groups were described in detail previously and are available in the protocols of the ADNI study (https://adni.loni.usc.edu/methods/). A total of 436 subjects (135 CN, 274 MCI, 27 AD) had 2‐year follow‐up Florbetapir‐PET scans available. A summary of demographic and cognitive characteristics for all clinical groups is displayed in Table 1.

**TABLE 1:** Sample characteristics

Clinical group	CN	MCI	AD
N	179	403	85
Age	73.8 ± 6.5	71.7 ± 7.7	75.6 ± 8.3
Sex (M/F)	88/91	220/183	49/36
MMSE	29.1 ± 1.2	28.1 ± 1.7	22.9 ± 2.0

*Note:* Average values are reported as mean ± *SD*.

Abbreviations: MMSE, mini‐mental state examination; M, male; F, female.

In addition, we included Florbetapir‐PET scans of 13 cognitively normal young controls (YC) to estimate region‐specific amyloid‐positivity thresholds. All YC subjects were younger than 45 years of age (mean ± *SD*: 27 ± 4.3 y), the sample had equal gender distribution (M/F: 6/7), and low APOE4 positivity prevalence (9 negative, 1 positive, 3 unknown). The data was originally acquired to serve as a YC reference data set for scaling of global Florbetapir‐PET signal to the standardized Centiloid scale (Navitsky et al., 2018a) and is freely available on the Global Alzheimer Association Interactive Network website (GAAIN; http://www.gaain.org/centiloid-project).

As reported previously (Grothe et al., 2017; Navitsky et al., 2018a), written informed consent was obtained for all study participants and/or authorized representatives, and data collection and sharing were performed in accordance with the ethical standards of the institutional and/or national research committees (http://adni.loni.usc.edu/methods/documents/) (Navitsky et al., 2018a). Only de‐identified imaging and clinical data was used for the analysis according to data sharing and publication policies of the providing institutions (http://adni.loni.usc.edu/data-samples/access-data/).

### 2.2 Imaging data

Detailed acquisition and standardized pre‐processing steps of the Florbetapir‐PET images are available at the ADNI website (adni.loni.usc.edu/methods/). Briefly, Florbetapir‐PET images for all participants were acquired as 4 × 5 min frames 50 to 70 min after 370 MBq bolus injection of Florbetapir (^18^F). To account for the multicentric acquisition of the data, all images undergo standardized pre‐processing steps within ADNI, including realignment and averaging of the dynamic volumes, reslicing to a common voxel size (1.5 mm × 1.5 mm × 1.5 mm), and scanner‐specific smoothing to an approximate final resolution of 8 mm FWHM. For further pre‐processing we also used the corresponding 3T T1‐weighted 3D magnetization‐prepared rapid acquisition gradient echo (MPRAGE) MRI scan that was closest in time to the Florbetapir‐PET scan (all obtained within 3 months from the corresponding PET scans; interval = 1.08 ± 1.06 months).

Pre‐processing and analysis of both baseline and follow‐up imaging data followed previously reported procedures (Grothe et al., 2017; Sakr et al., 2019). Briefly, MRI images were realigned, segmented into tissue types, and spatially normalized to an aging/AD‐specific reference template space using standard procedures (Ashburner, 2007) implemented in Statistical Parametric Mapping software (SPM8, the Wellcome Trust Centre for Neuroimaging). Florbetapir‐PET images were rigidly aligned to their corresponding structural MRI scan in native space, corrected for partial volume effects (PVE) using the “Müller‐Gärtner” method (Gonzalez‐Escamilla et al., 2017; Müller‐Gärtner et al., 1992), and spatially normalized to the reference template space using transformation parameters from the corresponding MRI. Fifty‐two regional volumes of interest (rVOI) were defined within the reference template using the Harvard‐Oxford atlas, including 48 different cortical regions, as well as the hippocampus, amygdala, striatum, and thalamus, all masked for >50% gray matter probability. Regional standard uptake value ratios (SUVR) for rVOI were calculated by scaling mean tracer uptake values to the mean uptake value of the whole cerebellum. The employed 3‐compartment PVE correction method (Müller‐Gärtner et al., 1992) has been shown to provide improved estimates of regional amyloid tracer binding in the brain's gray matter (Gonzalez‐Escamilla et al., 2017; Rullmann et al., 2016), but it does not provide values for the white matter compartment. Thus, the signal of the whole cerebellar reference region (including cerebellar gray and white matter) that is most commonly used for scaling Florbetapir‐PET data (Clark et al., 2012; Navitsky et al., 2018a) was extracted from the PET data before PVE correction (Grothe et al., 2017).

Adopting a commonly used approach for defining global signal cut‐offs (Jack Jr et al., 2017; Joshi et al., 2012), regional amyloid‐positivity within each rVOI was defined as 1.65 SDs above the mean value in the YC sample. Acquisition parameters for the YC imaging data followed similar protocols as for the ADNI data and are detailed in the original publication of this dataset (Navitsky et al., 2018a). Pre‐processing and analysis of Florbetapir‐PET images of the YC group followed the identical MRI‐based processing pipeline as described above. Given the relatively small sample size, mean and *SD* values for all rVOIs were calculated on 10,000 bias‐corrected and accelerated bootstrap resamples. In sensitivity analyses we used alternative cut‐off definitions based on 2 and 3 SDs above the mean value, as well as using the maximum value observed in the YC sample.

For comparison, we also studied standard global cortical SUVRs (with whole cerebellum reference) for all ADNI PET scans using centrally calculated values that are made available on the ADNI server (https://adni.loni.usc.edu/methods/pet-analysis). Global amyloid‐positivity was defined using a recommended cut‐off of SUVR >1.1 (Joshi et al., 2012; Landau et al., 2014).

### 2.3 Cerebrospinal fluid (CSF) AD biomarkers

Amyloid‐β 1–42 peptide (Aβ_1‐42_), total tau (t‐tau), and phosphorylated tau (p‐tau) in the CSF were measured using the multiplex xMAP Luminex platform with Innogenetics immunoassay kit–based reagents. Immunoassay reagents, analytical platform, and other details of biomarker quantification are described elsewhere (Shaw et al., 2009). 90% of the included ADNI sample in this study (603 subjects) had complete baseline CSF biomarker data measured at the same study point as the neuroimaging data.

### 2.4 Cognitive assessment

Cognition was assessed using the mini‐mental state examination (MMSE) (Folstein, Folstein, & McHugh, 1975) as a measure of global cognitive performance, as well as 30‐min delayed recall (DR) of the Rey Auditory Verbal Learning Test (Schoenberg et al., 2006) and the Trail Making Test B (TMT‐B) (Reitan, 1958) as domain‐specific measures of episodic memory and executive function, respectively.

### 2.5 Cross‐sectional model of regional amyloid progression and staging scheme

Estimation of a regional amyloid progression model and derived staging scheme followed the same general approach as in our previous staging study (Grothe et al., 2017), including calculation of regional frequencies of amyloid‐positivity in the CN cohort (percentage of individuals showing suprathreshold SUVR in the different rVOI) as an indicator of regional pathology progression, and grouping single regions into four larger anatomical divisions based on their estimated temporal involvement (frequency count). Methodological improvements over the original approach included the calculation of regional amyloid‐positivity based on region‐specific cut‐offs as determined in the YC data, as well as defining the four larger anatomical divisions using the optimal k‐means clustering algorithm (Wang & Song, 2011) instead of equal partitions of the observed amyloid‐positivity frequency range. Moreover, in sensitivity analyses we assessed potential effects of confounding factors on the estimated regional progression pattern by repeating the analysis in separate subsamples of males and females, APOE4 carriers and non‐carriers, older and younger individuals (using a median split at 73y), as well as higher and lower educated individuals (median split at 16 years of education). The similarity of the progression sequences derived from the different subgroups was assessed by calculating pair‐wise Spearman rank correlations between the respective regional frequency counts (Grothe et al., 2017).

Following the previously described protocol for individual amyloid staging analyses (Grothe et al., 2017), each anatomical division was considered positive for amyloid pathology if at least 50% of rVOI within this division were displaying suprathreshold signal. The individual in‐vivo amyloid stage was then assigned based on the estimated hierarchical involvement of anatomical divisions. For example, stage I was defined as being positive in the first anatomical division, but negative in all following divisions, and stage II as being positive in first and second anatomical division, but not in third or fourth. Individual pathology profiles that would not follow the hierarchical order of anatomical divisions as predicted by the estimated staging scheme were considered non‐stageable (mismatch). Individual profiles that would not display suprathreshold signal in at least 50% of rVOI within any anatomical division were assigned stage 0. Given that CN subjects were also used to derive the regional progression model, an unbiased estimate of non‐stageable CN subjects was calculated using 10,000 resamples of randomly selected test and train subsamples. In sensitivity analyses, we assessed staging outcomes when systematically altering both the rVOI positivity requirement for an anatomical division to be considered positive (initially set to 50%) as well as the overall number of considered anatomical divisions (initially set to 4).

### 2.6 In‐vivo stages and longitudinal amyloid progression

To estimate the validity of the cross‐sectionally estimated amyloid progression model, we studied regional amyloid progression using 2‐year follow‐up imaging data. First, in order to validate the cross‐sectional in‐vivo staging approach, we determined the in‐vivo amyloid stages for the follow‐up dataset and assessed how many subjects progressed, remained stable, or displayed unexpected progression patterns (reverse stages or progression along deviating patterns).

In addition to the longitudinal staging analyses, we also explored the temporal progression of regional amyloid‐positivity independently of the cross‐sectionally estimated staging scheme. For this we used a probabilistic model that starts with the longitudinal detection of earliest amyloid accumulation in cases that are amyloid‐negative at baseline, and then iteratively searches for individuals at increasing amyloid progression phases in the baseline data to record their regional progression over follow‐up. Thus, based on the longitudinal rVOI changes in the subset of subjects that displayed no suprathreshold signal in any of the rVOI at baseline, we defined a regional profile corresponding to the first signs of amyloid pathology deposition. This profile was then used to select another subset of subjects who already showed the corresponding rVOI positivity profile at baseline. Assuming that these subjects were at the second year of the progression from an all‐negative rVOI state, we analyzed their follow‐up data to define a profile for an estimated fourth year of amyloid pathology progression. In total, this procedure was repeated until the remaining individual profiles at baseline would display amyloid‐positivity in more than 70% of analyzed rVOI, indicating globally widespread amyloid pathology. To allow independent validation against our cross‐sectional model, we excluded from this analysis all baseline regionally positive CN individuals that were also used to define the cross‐sectional model (N = 116). The CN individuals who were all‐negative at baseline did not contribute to the estimated progression sequence in the cross‐sectional model (based on regional positivity counts) and were thus included in the longitudinal probabilistic analysis. The obtained results were used to approximate amyloid pathology progression for approximately 10 years from an all‐negative baseline state. For each of the analysis steps, the frequency of rVOI amyloid‐positivity in follow‐up data was used to calculate the probability of a region to become involved after each of the modeled 2‐year intervals. Note that assignment of “progression year” in the model is a modeled approximation that does not necessarily correspond to actual individual timelines of amyloid pathology progression.

### 2.7 Statistical analyses

Cross‐sectional associations of in‐vivo amyloid stages with baseline CSF AD biomarkers and cognitive test scores were assessed using age‐corrected Spearman rank correlation coefficients (ρ). In addition, for each amyloid stage differences in baseline CSF biomarker values were compared to its preceding stage using Mann–Whitney U tests. Similarly, in the longitudinal staging analysis Mann–Whitney U tests were used to compare baseline CSF biomarker values between groups that remained stable in their respective stage and those who progressed to a different stage at follow‐up. Differences in the distribution of baseline in‐vivo amyloid stages between clinical groups were evaluated using Pearson's Chi‐squared test (χ^2^).

## 3 RESULTS

### 3.1 Updated cross‐sectional staging model of regional amyloid progression

**FIGURE 1:**
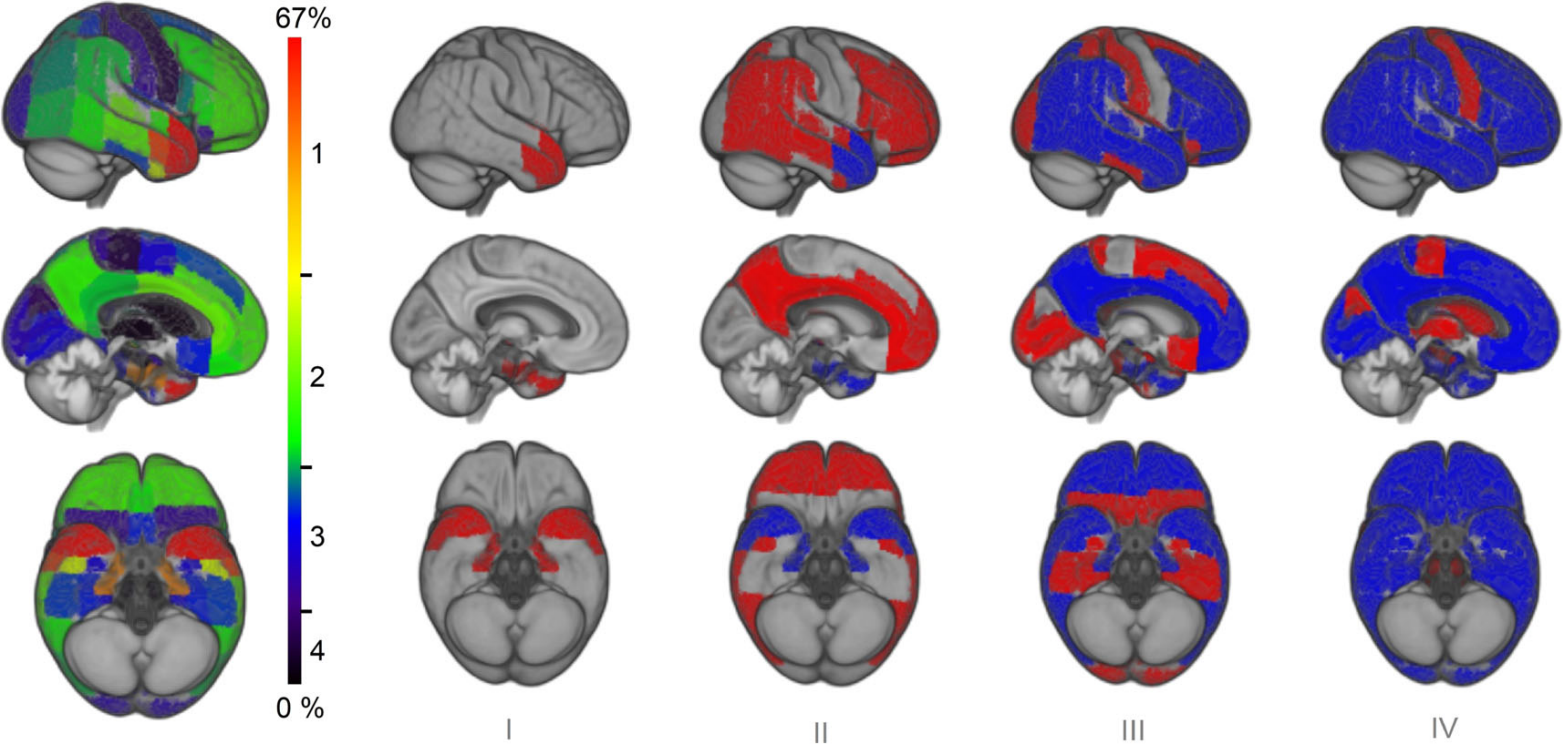
Four‐stage model of regional amyloid progression. Brain renderings on the left illustrate the frequency of regional amyloid‐positivity across individuals on a color scale from black/blue (lowest) to yellow/red (highest). In the resulting four‐stage model of regional amyloid progression (I–IV), incremental stages are defined by involvement of higher numbered anatomic divisions (in red), in addition to the affected areas of the previous stage (blue)

As expected, regional means and *SD*s of SUVR values in the YC data differed considerably between rVOIs, resulting in variable region‐specific amyloid‐positivity cut‐offs (Supplemental Figure 1). Applying these cut‐offs to the regional SUVRs in the CN cohort resulted in a frequency‐based progression model (Figure 1 and Supplemental Figure 2) where most frequent involvement was observed for the temporal pole (67%), anterior parts of the middle temporal (62%) and parahippocampal gyri (56%), and the planum polare (51%) (stage I). The anterior temporal lobe regions were followed by widespread frontal, temporal, and parietal association regions (40–20%), including for example, the anterior inferior temporal gyrus (39%), anterior (32%) and posterior (23%) cingulate cortex, and precuneus (28%) (stage II). A third stage involved posterior temporobasal and medial temporal areas, as well as most parts of the occipital cortex and the postcentral gyrus (20%–10%), whereas the final stage was characterized by amyloid deposition in the precentral cortex, hippocampus, and striatum (10–0%). Regional progression models estimated in subsamples stratified by demographic (age, gender, education) or genetic (APOE4) variables were all highly similar to the original model calculated in the whole CN group (all pair‐wise Spearman rank correlation coefficients ρ > .96, *p* < .001), indicating little effect of these variables on the regional distribution of amyloid‐positivity (Supplemental Figure 3). Moreover, regional progression models calculated for varying cut‐off definitions all showed a highly similar ordering of the 52 brain regions (all pair‐wise Spearman rank correlation coefficients ρ > .96, *p* < .001; Supplemental Figure 4).

**FIGURE 2:**
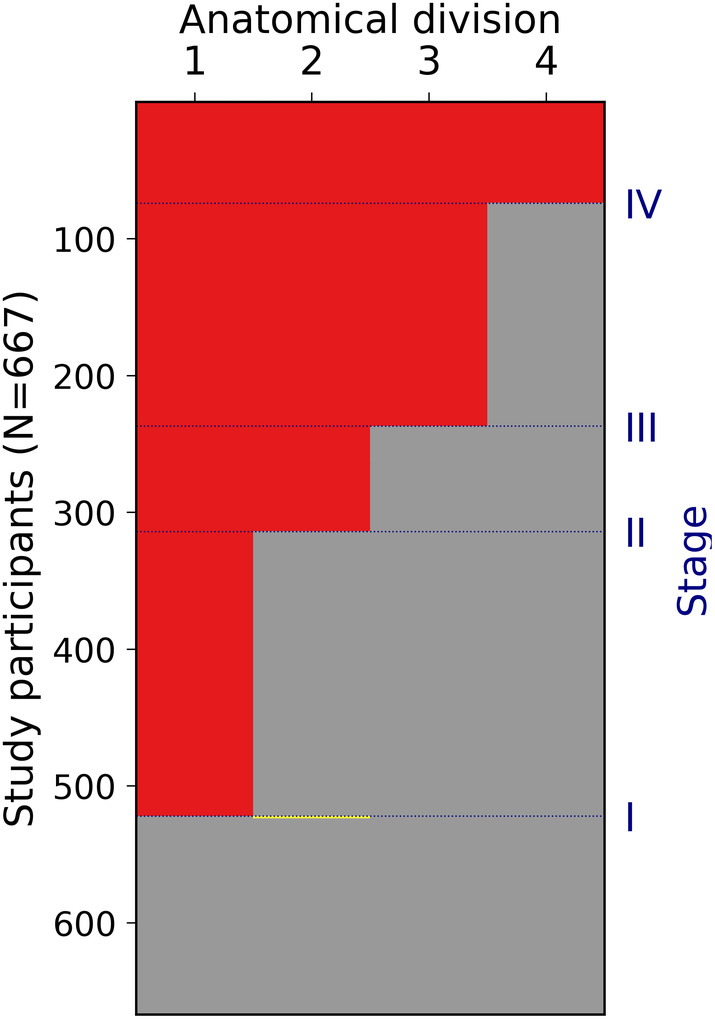
Staging of individual amyloid burden. Six hundred and sixty‐seven study participants are plotted as rows of the matrix, anatomical divisions are plotted as columns. Absence or presence of amyloid is displayed in gray and red, and two subjects that deviated from the predicted pattern are displayed in yellow (1 CN and 1 MCI)

Amyloid‐positivity in the four anatomical divisions defining the cross‐sectional staging scheme showed a highly consistent regional hierarchy across individual amyloid deposition profiles (Figure 2). Only 1% (2 individuals) of the amyloid‐positive subjects in the analyzed sample and on average only 2 ± 4% of the CN subjects in the sensitivity analyses displayed non‐stageable patterns. Sensitivity analyses altering the total number of considered anatomical divisions and the methodology to define positivity for each anatomic division indicated that the a priori settings (4 divisions, 50% positivity requirement) offer a good trade‐off for sensitive detection of regional amyloid‐positivity in a relatively detailed staging model while keeping the number of unstageable subjects as low as possible (Supplemental Figure 5).

Stage I subjects were mostly classified as amyloid‐negative according to standard global cortical SUVRs but displayed significantly lower CSF‐Aβ_1‐42_ values as compared to stage 0 (−6.6%, *p* < .001) (Table 2), and overall, higher amyloid stages were strongly associated with lower CSF‐Aβ_1‐42_ levels (ρ = −.72, *p* < .001). Additionally, higher amyloid stages were associated with higher t‐tau (ρ = .52, *p* < .001) and p‐tau (ρ = .56, *p* < .001) levels. In pairwise comparisons, both t‐tau and p‐tau levels were significantly higher in stage II compared to stage I and stage 0, but did not differ between stage 0 and stage I (Table 2). No significant CSF‐based amyloid or tau biomarker differences were found between stage III and stage IV, probably reflecting saturation effects in these measures (Jack Jr. et al., 2012; Mattsson et al., 2015).

**TABLE 2:** Amyloid stages in comparison to clinical diagnosis, CSF biomarkers, and global Florbetapir‐PET signal

	Stage 0	Stage I	Stage II	Stage III	Stage IV
N	143	208	77	163	74
CN	35.7%	40.9%	20.8%	15.3%	1.4%
MCI	59.4%	57.7%	68.8%	63.2%	55.4%
AD	4.9%	1.4%	10.4%	21.5%	43.2%
Aβ_1‐42_	227 ± 32	212 ± 40*	142 ± 32*	132 ± 22**	127 ± 19
t‐tau	57.3 ± 21.6	61.7 ± 33.1	97 ± 52.4*	123.3 ± 54*	122.1 ± 59.7
p‐tau	29.7 ± 15	28.5 ± 14.2	44 ± 22.5*	57.7 ± 24.6*	58.5 ± 29.3
SUVR	0.99 ± 0.06	1.04 ± 0.07*	1.22 ± 0.09*	1.41 ± 0.12*	1.55 ± 0.14*
SUVR>1.1	6%	15%	88%	100%	100%
Higher stage at 2y follow‐up	21.7%	8.2%	18.2%	11%	—
Annual SUVR change	0.9 ± 1.9%	3.6 ± 2.6%	4.6 ± 3.9%	4.2 ± 4.7%	—

*Note:* Values are reported as mean ± *SD*.

Abbreviation: SUVR, global cortical standard uptake value ratio.

* Significantly different from the previous stage at *p* ≤ .001, Mann–Whitney *U* test.

** Significantly different from the previous stage at *p* ≤ .01, Mann–Whitney *U* test.

**FIGURE 3:**
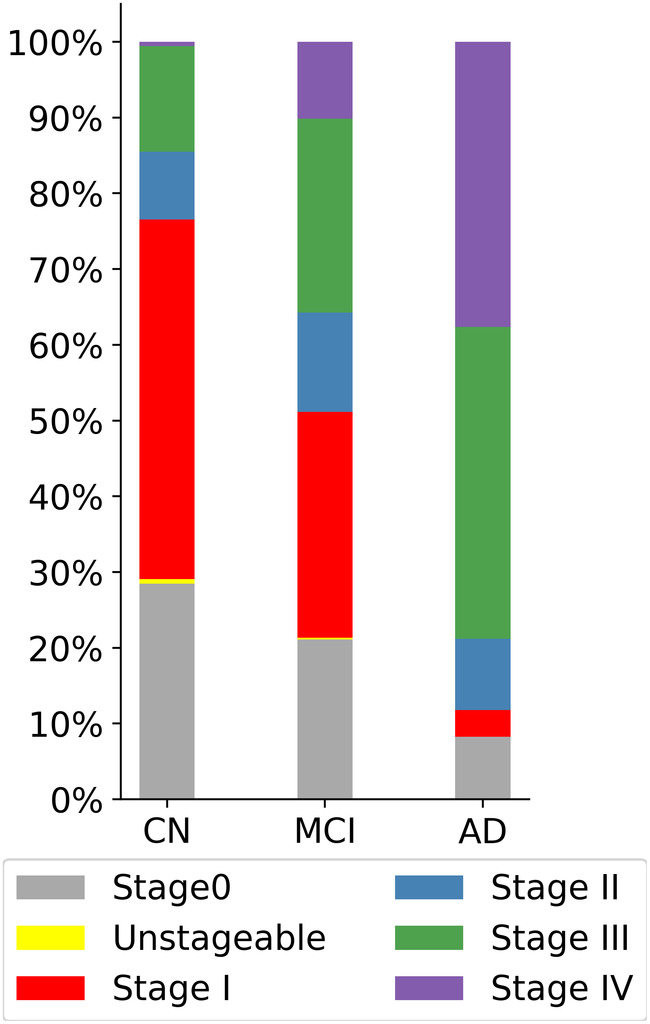
Proportions of in vivo amyloid stages by clinical diagnosis. Higher amyloid stages are more likely to be observed in the MCI and AD groups. Only one subject in the CN group was classified as stage IV

Distribution of in‐vivo amyloid stages differed significantly between clinical groups (χ2 = 117.66, *p* < .001; Figure 3). Advanced amyloid stages III and IV accounted for 86% of amyloid deposition profiles in AD, 45% in MCI, and 20% in the CN group, with only a single CN subject classified as stage IV. Within separate clinical groups, higher amyloid stages (I‐IV) were associated with lower DR scores in the CN, MCI, and AD groups, as well as with higher TMT‐B scores and lower MMSE scores in the MCI group (Table 3). Similar associations were observed for standard global cortical SUVR values (> 1.1), although associations with DR in the CN group did not reach statistical significance.

**TABLE 3:** Associations between amyloid severity and cognitive scores

Cognitive test	CN	MCI	AD
In‐vivo amyloid stages			
MMSE, AU	ρ_128_ = −.11, *p* = .22	ρ_318_ = −.23, *p* < .001	ρ_78_ = −.13, *p* = .26
DR, AU	ρ_128_ = −.19, *p* = .03	ρ_313_ = −.36, *p* < .001	ρ_77_ = −.25, *p* = .03
TMT, sec	ρ_128_ = .024, *p* = .78	ρ_308_ = .2, *p* < .001	ρ_69_ = .16, *p* = .2
Global SUVR (>1.1)			
MMSE, AU	ρ_53_ = −.06, *p* = .65	ρ_219_ = −.21, *p* = .002	ρ_75_ = .08, *p* = .51
DR, AU	ρ_53_ = −.2, *p* = .14	ρ_215_ = −.29, *p* < .001	ρ_74_ = −.23, *p* = .05
TMT, sec	ρ_53_ = .01, *p* = .93	ρ_214_ = .28, *p* < .001	ρ_67_ = −.02, *p* = .87

Abbreviations: MMSE, mini‐mental state examination; DR, delayed recall; TMT, Trail Making Test B.

### 3.2 Longitudinal staging of individual amyloid progression

**FIGURE 4:**
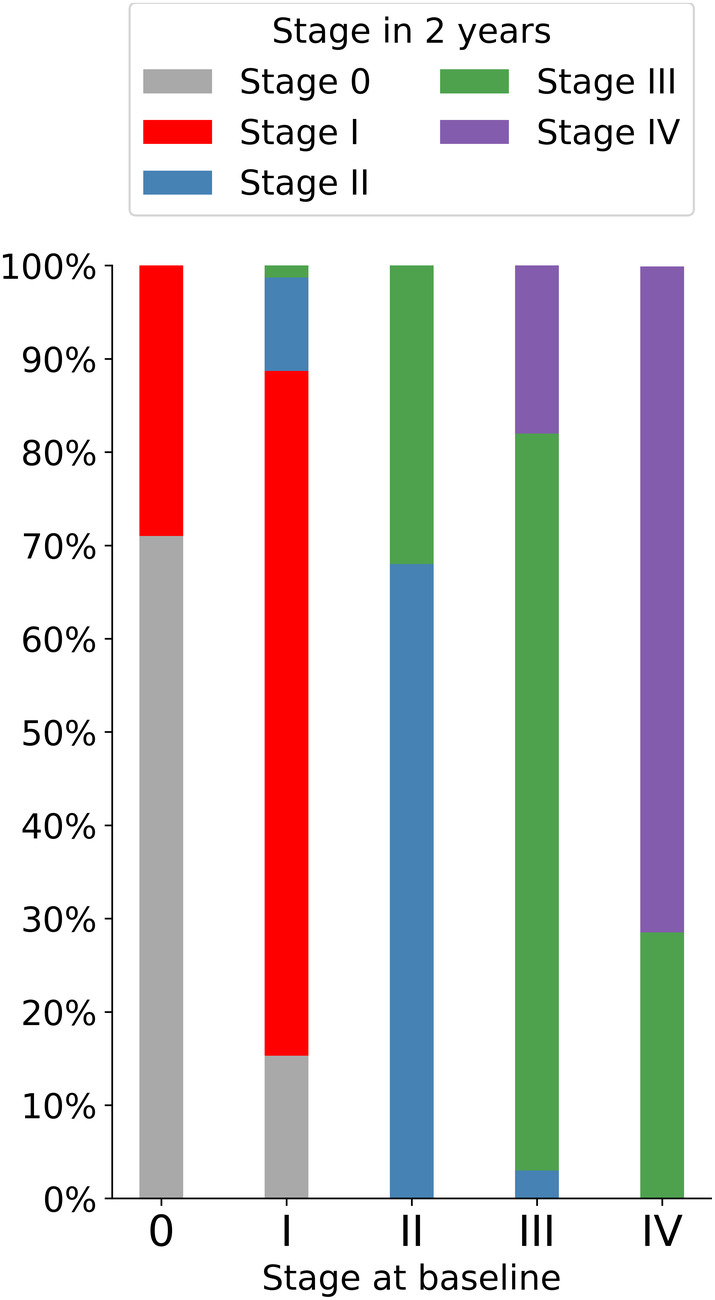
Amyloid stages in 2‐year follow‐up data. Bar plots display the stage at baseline, with the proportion of stages in 2‐year follow up data displayed by color code

Out of 436 subjects that had 2‐year follow‐up imaging data available, 73.4% of subjects remained at the same stage after two years, 18.3% showed a model‐conform transition to a higher stage, 8.3% decreased to a lower stage, and no subjects followed a regional progression patterns that deviated from the staging model. Reverse progressions almost entirely corresponded to reclassifications of stage I to stage 0, as well as stage IV to stage III at follow‐up, affecting 15.3% of stage I and 28.5% stage IV individuals at baseline. Distribution of the stages in the 2‐year follow‐up data by the stage at baseline is displayed in Figure 4.

A Mann–Whitney test indicated that there was no significant difference in the baseline CSF‐Aβ_1‐42_ values of the stage 0 subjects that progressed to stage I (228 ± 31) over follow‐up compared to the baseline CSF‐Aβ_1‐42_ values of those who remained stable at stage 0 (227 ± 34) (U = 1,149, *p* = .38). Additionally, the baseline CSF‐Aβ_1‐42_ values of stage I subjects that reversed to stage 0 (227 ± 39) at follow‐up were not different from the baseline CSF‐Aβ_1‐42_ values of the stage 0 subjects (227 ± 34) (U = 1,621, *p* = .46). By contrast, stage I subjects that remained stable over two years had significantly lower baseline CSF‐Aβ_1‐42_ values (215 ± 40) as compared to the baseline CSF‐Aβ_1‐42_ values of the stage I subjects that reversed to stage 0 (227 ± 39) (U = 982.5, *p* = .047), and significantly higher baseline values as compared to the subjects that transitioned from stage I to stages II or III over follow‐up (179 ± 27) (U = 614, *p* = .01).

### 3.3 Probabilistic longitudinal trajectory model

**FIGURE 5:**
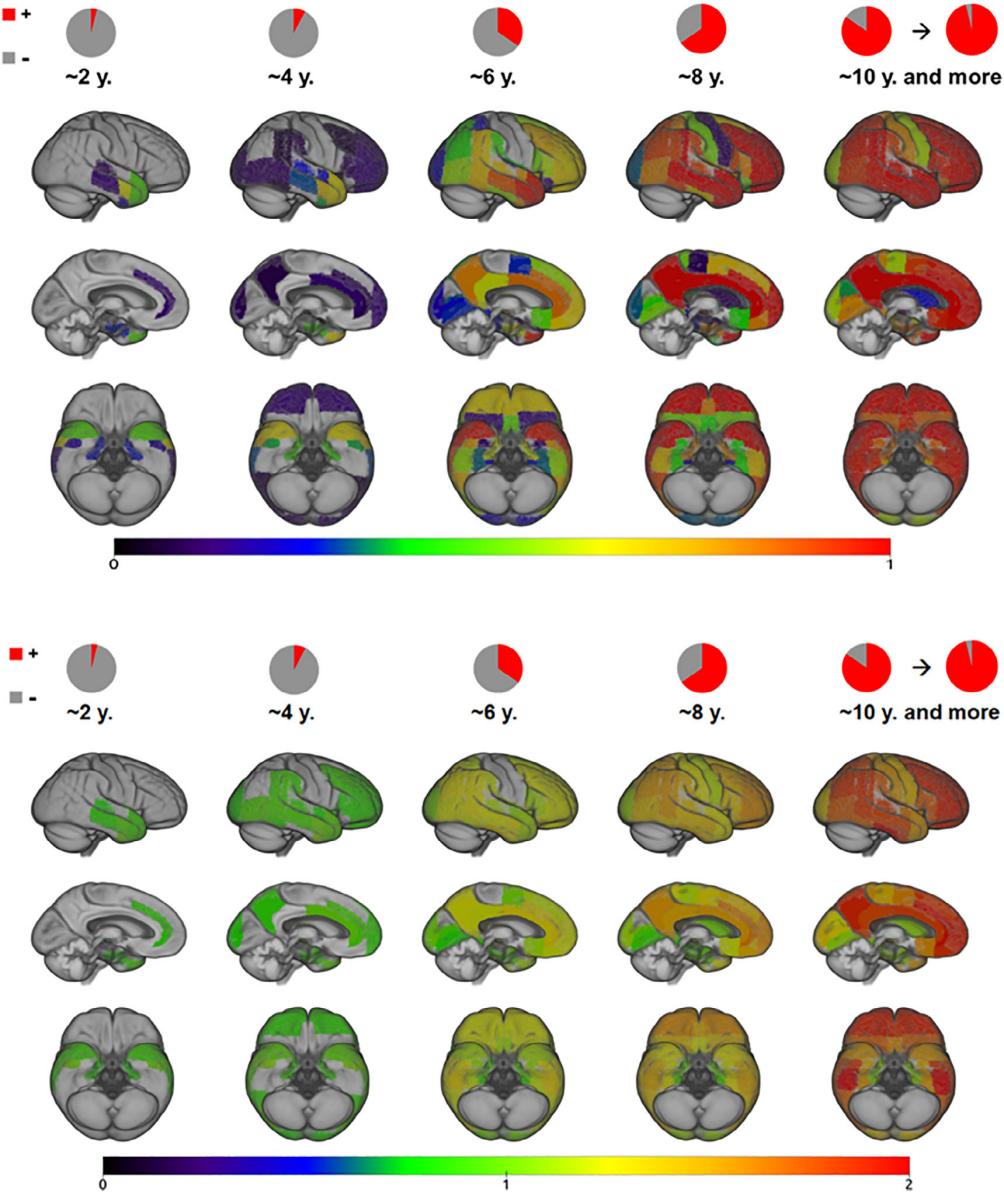
The probabilistic longitudinal trajectory model. Progression phases are modeled for 2‐year intervals from an all‐negative state. In the upper panel warmer color corresponds to a higher probability (*p* ≥ 5%) of the region to become amyloid‐positive. The lower panel displays mean SUVR values at the different modeled time‐points for each of the regions displayed above. Pie charts display the average amyloid‐positive region count at each of the modeled time points

16% of the selected subcohort with follow up data (N = 46/320) displayed no suprathreshold signal in any of the rVOI at baseline. Analysis of 2‐year follow‐up images of these subjects suggested a very similar regional pattern of earliest amyloid deposition as the one estimated by the cross‐sectional frequency‐based model (Figure 5 and Supplemental Figure 6). Thus, first longitudinal changes from an all‐negative state most likely occurred in stage I regions, including the temporal pole and anterior parts of the middle temporal and parahippocampal gyri. This was followed by increasing probability of stage II regions to become involved, whereas probability for stage III regions to become positive began to increase from modeled progression year 6 onwards. Stage IV regions were highly unlikely to become positive before the modeled progression years 8–10.

Similarly to the CSF‐Aβ_1‐42_ results for the longitudinal staging analysis, a Mann–Whitney test indicated that there was no significant difference in baseline CSF‐Aβ_1‐42_ values between all‐negative subjects who remained negative (230 ± 23) and those who showed regional amyloid‐positivity at 2‐year follow‐up (228 ± 39) (U = 242, *p* = .32).

## 4 DISCUSSION

In the present study we have reassessed our previously developed in‐vivo amyloid staging model (Grothe et al., 2017) by applying new methodology that accounts for regionally varying noise levels in the Florbetapir‐PET imaging data. Additionally, we evaluated the validity of the cross‐sectionally estimated amyloid progression model using longitudinal PET data. Similarly to the previous model (Grothe et al., 2017) and to the established neuropathologic amyloid staging schemes (Braak et al., 2011; Braak & Braak, 1991; Thal et al., 2002), the updated model shows a consistent pattern of spreading from associative neocortex over allocortical and primary sensory‐motor areas to subcortical structures. Compared to our previous staging model, the use of regionally varying amyloid‐positivity cut‐offs altered the temporal ordering of some neocortical regions, but overall resulted in a similar progression pattern across macroscopic brain systems as well as largely identical amyloid staging characteristics across individuals. Longitudinal progression analyses demonstrated that individual progression patterns closely match the cross‐sectionally estimated staging scheme.

In the cross‐sectional progression model, the greatest effect of the regionally varying thresholds is observed for the chronological ordering of specific temporal lobe regions, shifting earliest amyloid accumulating regions from inferior temporal to anterior temporal lobe areas. An early increase of amyloid signal in the temporal neocortex has been reported in several previous studies on independent study cohorts employing Florbetapir (Gonneaud et al., 2017; Rodrigue et al., 2012) or Florbetaben radiotracers (Cho et al., 2016). The results were also supported by our longitudinal progression analysis in completely amyloid‐negative subjects at baseline, who after 2 years of follow‐up displayed amyloid‐positivity in the same anterior temporal lobe regions (corresponding to stage I of the cross‐sectional staging scheme). Nonetheless, these results are in contrast with reports of early amyloid accumulation in anterior and posterior midline regions (Mattsson, Palmqvist, Stomrud, Vogel, & Hansson, 2019; Palmqvist et al., 2017; Villeneuve et al., 2015). Such differences can most likely be explained by the different analytic approaches across studies. Thus, studies reporting an early involvement of the temporal neocortex typically analyzed direct signal elevations across individuals (Cho et al., 2016; Gonneaud et al., 2017; Rodrigue et al., 2012), whereas elevated amyloid‐PET signal in anterior and posterior midline regions is principally observed in studies contrasting high‐amyloid groups to age‐matched low‐amyloid control groups (Mattsson et al., 2019; Palmqvist et al., 2017). Moreover, previous longitudinal amyloid‐PET studies have typically focused on spatiotemporal dynamics as reflected by regional accumulation rates of SUVR values (Bilgel, Prince, Wong, Resnick, & Jedynak, 2016; Marinescu et al., 2019; Mattsson et al., 2019; Villain et al., 2012), whereas the focus of our longitudinal modeling approach lied on the regional order of amyloid progression as indicated by the occurrence of suprathreshold SUVR signal (i.e., regional amyloid‐positivity), which is the metric used by the regional staging approach. We note that these two metrics, occurrence of suprathreshold SUVR signal and SUVR accumulation rates, provide complementary information on the regional onset of pathology and its further temporal dynamics, respectively. Interestingly, a comparison of the evolution of mean regional SUVR values with the probabilistic information on regional amyloid‐positivity in our longitudinal probabilistic model suggests that the observed regional onset of pathology does not coincide with fastest progression in amyloid accumulation (Figure 5). Thus, SUVR values of the temporal regions identified to exhibit the first suprathreshold signal displayed only a rather moderate increase over the modeled time intervals, and were surpassed at later time points by the higher SUVR increases in AD‐typical frontoparietal regions that were also consistently identified as fast accumulating regions in previous longitudinal amyloid‐PET studies (Bilgel et al., 2016; Marinescu et al., 2019; Mattsson et al., 2019; Villain et al., 2012).

Gonneaud et al. (2017) suggested that the early signal increase in the temporal neocortex may be attributable to “physiologic” age‐related amyloid accumulation that occurs independently from AD‐related changes in the anterior and posterior midline regions. However, these early signal elevations could also reflect some form of (age‐related) off‐target binding, or a confound by blood flow and radiotracer delivery effects as previously described for settings with low global amyloid levels (Sojkova et al., 2015). Here we find that the regionally restricted temporal lobe signal defining stage I in our study corresponds to lower CSF‐Aβ_1‐42_ values (but not different CSF tau values) as compared to stage 0, which suggests that this signal at least partly represents amyloid accumulation. The initial signal increase in anterior temporal lobe regions is followed by amyloid‐positivity in several neocortical association areas that have previously been linked to early AD‐related amyloid accumulation (together defining stage II of our staging scheme) (Gonneaud et al., 2017; Mattsson et al., 2019; Palmqvist et al., 2017; Villeneuve et al., 2015). Interestingly, we also found that, in addition to a further decrease in CSF‐Aβ values, involvement of these stage II regions was associated with a significant increase in tau biomarker values, thus corroborating their closer link to AD‐related pathology. Nonetheless, the exact nature of early signal increases in the temporal neocortex and their significance for AD‐related pathologic changes remains to be studied in more detail. To date all amyloid‐PET studies reporting early signal increases in the temporal neocortex employed ^18^F‐based amyloid radiotracers (Cho et al., 2016; Gonneaud et al., 2017; Rodrigue et al., 2012), and thus studying these regional signal elevations using ^11^C‐based PIB‐PET may provide additional information as to the tracer‐specificity of this finding. For instance, tracers may differ in their binding characteristics to specific types of amyloid‐β aggregates, potentially also resulting in differential binding affinities to the early “diffuse” and later “neuritic” forms of amyloid plaques (Beach et al., 2018; Ni, Gillberg, Bergfors, Marutle, & Nordberg, 2013; Seo et al., 2017).

In the longitudinal staging analysis, none of the progressed individuals deviated from the regional progression pattern predicted by the cross‐sectional staging model. However, a relatively large proportion of individuals classified as stage I at baseline showed an unexpected regression to stage 0, indicating that these early signal elevations may be more susceptible to false positive errors. Accordingly, in contrast to stage I individuals that stayed stable or further progressed, those who reverted to stage 0 did not show lower CSF‐Aβ_1‐42_ levels at baseline compared to stage 0 individuals. Interestingly, baseline CSF‐Aβ_1‐42_ levels were lower in progressive compared to stable stage I individuals. This agrees with previous reports of CSF‐Aβ_1‐42_ changes preceding global amyloid accumulation (Palmqvist et al., 2017). However, we did not observe any evidence for lower CSF‐Aβ_1‐42_ levels preceding the early amyloid accumulation in stage I regions (i.e., no difference between all‐negative individuals who progressed vs those who remained stable).

When characterizing amyloid progression by longitudinal change in global cortical Florbetapir‐PET values, individuals who progressed from stage 0 to stage I showed only a small annual increase of +0.9% (Table 2), which is expected based on the subtle, spatially circumscribed signal elevations that define stage I. By contrast, mean annual global SUVR increase for individuals progressing to cortically more widespread stages II‐IV was in the range of 3.6–4.6%. For comparison, a previous longitudinal Florbetapir‐PET study using ADNI data reported mean annual increases in global SUVR values (with whole cerebellar reference) of 1.4 ± 3.9% (range: −9.3% to +15%) in the early phase of amyloid progression as defined by CN and early MCI individuals with a positive amyloid‐status defined by CSF Aβ_1‐42_ values (Landau et al., 2015). Thus, mean global SUVR changes in our study are well in the range of this previous study, and indicate increased global cortical amyloid accumulation from stage I onwards.

The data‐driven probabilistic longitudinal trajectory model revealed a pattern of regional amyloid spread that was highly consistent with the pattern estimated by regional frequency counts in the cross‐sectional staging scheme, thus supporting the validity of this staging approach as a method for estimating an individual's pathology progression on the basis of a single cross‐sectional PET scan. This method may be particularly useful for risk stratification in preclinical cohorts, where the utility of clinical data for staging disease progression is very limited. Importantly, increasing amyloid stages were also associated with lower memory scores among cognitively normal individuals, thus indicating the potential of this method for estimating risk of disease progression towards clinically relevant stages during the preclinical phase of AD. Recent studies of the topography of amyloid progression suggest that amyloid spread to subcortical regions is associated with higher risk of cognitive decline among nondemented individuals (Cho et al., 2018; Hanseeuw et al., 2018). Hanseeuw et al. (2018) suggested to stratify subjects into three stages according to their cortical and striatal radiotracer uptake values (low cortical, high cortical, and high cortical with high striatal signal), and showed that high neocortical and striatal signal predicted faster cognitive decline than elevated cortical signal alone. A similar staging approach was employed by Cho et al. (2018), who observed cognitive changes over a decade‐long period and reported that involvement of subcortical regions (amygdala and striatal regions) was associated with the steepest longitudinal changes in cognitive measures. Our data‐driven staging system very similarly places striatum and other subcortical structures to the latest stage of regional amyloid accumulation but additionally offers a more detailed subdivision of the preceding cortical deposition stage. Farrell et al. (2018) suggested that earliest amyloid‐related memory decline among cognitively normal individuals most closely corresponds to regional amyloid‐PET signal increases in the medial and lateral parietal neocortex (corresponding to stage II of our regional staging scheme). Consistently, using long‐term clinical follow‐up information of up to 6 years we could most recently demonstrate that an individual's in‐vivo amyloid stage as defined by our four‐stage model translates into a stage‐proportional risk for cognitive decline, where cognitively normal individuals from stage II onwards were at a significantly increased risk for conversion to MCI compared to stage 0 (Teipel et al., 2020).

### 4.1 Methodological considerations

Our regional analysis of Florbetapir‐PET data from young healthy adults indicates that even in this assumedly amyloid‐negative group Florbetapir‐PET signal varies significantly across brain regions, thus strongly arguing for the use of region‐specific amyloid‐positivity cut‐offs. However, the derived cut‐offs in the present study are limited to a relatively small sample of young controls with available Florbetapir‐PET data (Navitsky et al., 2018a), and larger samples of truly amyloid‐negative individuals would be desirable for a more robust estimate of regional amyloid‐positivity thresholds. In our regional amyloid staging approach, we apply partial volume effect correction to the Florbetapir‐PET images, which has previously been shown to improve amyloid‐PET quantification (Brendel et al., 2015; Gonzalez‐Escamilla et al., 2017; Rullmann et al., 2016; Su et al., 2016), particularly by accounting for confounding effects of high nonspecific white matter binding (Matsubara et al., 2016). Nonetheless, quantitative amyloid imaging results are also affected by the choice of several other experimental variables, including type of acquisition, preprocessing techniques, and reference tissue selection (Landau et al., 2014; Schmidt et al., 2015). Thus, future methodological work should also assess potential effects of other commonly applied reference regions (Landau et al., 2015; Su et al., 2016) or of different approaches to PET signal quantification (e.g., full kinetic modeling instead of the simplified SUVR approach [Ottoy et al., 2017]). Finally, our longitudinal modeling of amyloid progression was limited to two time points with a relatively short interval of 2 years, which previously was associated with less stable amyloid trajectories than models based on three or more time points (Landau et al., 2018). Observing baseline amyloid‐negative subjects over longer intervals would result in more robust temporal amyloid progression estimates with less reliance on modeling assumptions and could also provide better insights into the long‐term stability of the observed regressions in early stage I subjects.

## 5 CONCLUSION

We have updated our previously published in‐vivo amyloid staging scheme to account for regionally varying noise levels in Florbetapir‐PET signal and provided evidence for the longitudinal validity of the cross‐sectionally estimated amyloid progression pattern. This technique may offer a valuable tool for stratification of pathologic disease progression based on a single cross‐sectional PET scan, with potentially important implications for early detection and risk assessment in the preclinical phase of AD. Further evaluation of the effects of regional amyloid progression on longitudinal dynamics of cognitive decline in at‐risk individuals is warranted.

## CONFLICT OF INTERESTS

S. J. T. participated in scientific advisory boards of Roche Pharma AG and MSD and received lecture fees from Roche and MSD. M. J. G. and I. J. have no conflict of interest to report.

## Supporting information


**Appendix** **S1**. Supporting Information

## Data Availability

The data that support the findings of this study were obtained from the Alzheimer’s Disease Neuroimaging Initiative (ADNI) database (http://adni.loni.usc.edu). De‐identified imaging and clinical data are shared without embargo through the LONI Image and Data Archive (IDA) upon approved data use application to the ADNI Data Sharing and Publications Committee (DPC). For more information please refer to http://adni.loni.usc.edu/data‐samples/access‐data/.

## References

[hbm25121-bib-0001] Ashburner, J. (2007). A fast diffeomorphic image registration algorithm. NeuroImage, 38(1), 95–113. 10.1016/j.neuroimage.2007.07.007 17761438

[hbm25121-bib-0002] Beach, T. G. , Maarouf, C. L. , Intorcia, A. , Sue, L. I. , Serrano, G. E. , Lu, M. , … Roher, A. E. (2018). Antemortem‐Postmortem correlation of Florbetapir (18F) PET amyloid imaging with quantitative biochemical measures of Aβ42 but not Aβ40. Journal of Alzheimer's Disease: JAD, 61(4), 1509–1516. 10.3233/JAD-170762 29376867

[hbm25121-bib-0003] Bilgel, M. , Prince, J. L. , Wong, D. F. , Resnick, S. M. , & Jedynak, B. M. (2016). A multivariate nonlinear mixed effects model for longitudinal image analysis: Application to amyloid imaging. NeuroImage, 134, 658–670. 10.1016/j.neuroimage.2016.04.001 27095307 PMC4912927

[hbm25121-bib-0004] Braak, H. , & Braak, E. (1991). Neuropathological stageing of Alzheimer‐related changes. Acta Neuropathologica, 82(4), 239–259. 1759558 10.1007/BF00308809

[hbm25121-bib-0005] Braak, H. , Thal, D. R. , Ghebremedhin, E. , & Del Tredici, K. (2011). Stages of the pathologic process in Alzheimer disease: Age categories from 1 to 100 years. Journal of Neuropathology and Experimental Neurology, 70(11), 960–969. 10.1097/NEN.0b013e318232a379 22002422

[hbm25121-bib-0006] Brendel, M. , Högenauer, M. , Delker, A. , Sauerbeck, J. , Bartenstein, P. , Seibyl, J. , … Alzheimer's Disease Neuroimaging Initiative . (2015). Improved longitudinal [(18)F]‐AV45 amyloid PET by white matter reference and VOI‐based partial volume effect correction. NeuroImage, 108, 450–459. 10.1016/j.neuroimage.2014.11.055 25482269

[hbm25121-bib-0007] Cho, H. , Choi, J. Y. , Hwang, M. S. , Kim, Y. J. , Lee, H. M. , Lee, H. S. , … Lyoo, C. H. (2016). In vivo cortical spreading pattern of tau and amyloid in the Alzheimer disease spectrum. Annals of Neurology, 80(2), 247–258. 10.1002/ana.24711 27323247

[hbm25121-bib-0008] Cho, S. H. , Shin, J.‐H. , Jang, H. , Park, S. , Kim, H. J. , Kim, S. E. , … Alzheimer's Disease Neuroimaging Initiative . (2018). Amyloid involvement in subcortical regions predicts cognitive decline. European Journal of Nuclear Medicine and Molecular Imaging, 45(13), 2368–2376. 10.1007/s00259-018-4081-5 29980831 PMC6439472

[hbm25121-bib-0009] Clark, C. M. , Pontecorvo, M. J. , Beach, T. G. , Bedell, B. J. , Coleman, R. E. , Doraiswamy, P. M. , … AV‐45‐A16 Study Group . (2012). Cerebral PET with florbetapir compared with neuropathology at autopsy for detection of neuritic amyloid‐β plaques: A prospective cohort study. Lancet Neurology, 11(8), 669–678. 10.1016/S1474-4422(12)70142-4 22749065

[hbm25121-bib-0010] Farrell, M. E. , Chen, X. , Rundle, M. M. , Chan, M. Y. , Wig, G. S. , & Park, D. C. (2018). Regional amyloid accumulation and cognitive decline in initially amyloid‐negative adults. Neurology, 91(19), e1809–e1821. 10.1212/WNL.0000000000006469 30305451 PMC6251600

[hbm25121-bib-0011] Folstein, M. F. , Folstein, S. E. , & McHugh, P. R. (1975). “Mini‐mental state”: A practical method for grading the cognitive state of patients for the clinician. Journal of Psychiatric Research, 12(3), 189–198. 10.1016/0022-3956(75)90026-6 1202204

[hbm25121-bib-0012] Gonneaud, J. , Arenaza‐Urquijo, E. M. , Mézenge, F. , Landeau, B. , Gaubert, M. , Bejanin, A. , … Chételat, G. (2017). Increased florbetapir binding in the temporal neocortex from age 20 to 60 years. Neurology, 89(24), 2438–2446. 10.1212/WNL.0000000000004733 29150540

[hbm25121-bib-0013] Gonzalez‐Escamilla, G. , Lange, C. , Teipel, S. , Buchert, R. , Grothe, M. J. , & Initiative, A.'s. D. N. (2017). PETPVE12: An SPM toolbox for partial volume effects correction in brain PET ‐ application to amyloid imaging with AV45‐PET. NeuroImage, 147, 669–677. 10.1016/j.neuroimage.2016.12.077 28039094

[hbm25121-bib-0014] Grothe, M. J. , Barthel, H. , Sepulcre, J. , Dyrba, M. , Sabri, O. , Teipel, S. J. , & Alzheimer's Disease Neuroimaging Initiative . (2017). In vivo staging of regional amyloid deposition. Neurology, 89(20), 2031–2038. 10.1212/WNL.0000000000004643 29046362 PMC5711511

[hbm25121-bib-0015] Hanseeuw, B. J. , Betensky, R. A. , Mormino, E. C. , Schultz, A. P. , Sepulcre, J. , Becker, J. A. , … Harvard Aging Brain Study . (2018). PET staging of amyloidosis using striatum. Alzheimer's & Dementia: The Journal of the Alzheimer's Association, 14(10), 1281–1292. 10.1016/j.jalz.2018.04.011 PMC6219621 29792874

[hbm25121-bib-0016] Jack, C. R., Jr. , Vemuri, P. , Wiste, H. J. , Weigand, S. D. , Lesnick, T. G. , Lowe, V. , … Knopman, D. S. (2012). Shapes of the trajectories of 5 major biomarkers of Alzheimer disease. Archives of Neurology, 69(7), 856–867. 10.1001/archneurol.2011.3405 22409939 PMC3595157

[hbm25121-bib-0017] Jack, C. R., Jr. , Wiste, H. J. , Weigand, S. D. , Therneau, T. M. , Lowe, V. J. , Knopman, D. S. , … Petersen, R. C. (2017). Defining imaging biomarker cut points for brain aging and Alzheimer's disease. Alzheimer's & Dementia: The Journal of the Alzheimer's Association, 13(3), 205–216. 10.1016/j.jalz.2016.08.005 PMC5344738 27697430

[hbm25121-bib-0018] Jack, C. R. , Knopman, D. S. , Jagust, W. J. , Petersen, R. C. , Weiner, M. W. , Aisen, P. S. , … Trojanowski, J. Q. (2013). Tracking pathophysiological processes in Alzheimer's disease: An updated hypothetical model of dynamic biomarkers. The Lancet Neurology, 12, 207–216. 10.1016/s1474-4422(12)70291-0 23332364 PMC3622225

[hbm25121-bib-0019] Josephs, K. A. , Murray, M. E. , Whitwell, J. L. , Tosakulwong, N. , Weigand, S. D. , Petrucelli, L. , … Dickson, D. W. (2016). Updated TDP‐43 in Alzheimer's disease staging scheme. Acta Neuropathologica, 131(4), 571–585. 10.1007/s00401-016-1537-1 26810071 PMC5946692

[hbm25121-bib-0020] Joshi, A. D. , Pontecorvo, M. J. , Clark, C. M. , Carpenter, A. P. , Jennings, D. L. , Sadowsky, C. H. , … Florbetapir F 18 Study Investigators . (2012). Performance characteristics of amyloid PET with florbetapir F 18 in patients with Alzheimer's disease and cognitively normal subjects. Journal of Nuclear Medicine: Official Publication, Society of Nuclear Medicine, 53(3), 378–384. 10.2967/jnumed.111.090340 22331215

[hbm25121-bib-0021] Kok, E. , Haikonen, S. , Luoto, T. , Huhtala, H. , Goebeler, S. , Haapasalo, H. , & Karhunen, P. J. (2009). Apolipoprotein E‐dependent accumulation of Alzheimer disease‐related lesions begins in middle age. Annals of Neurology, 65(6), 650–657. 10.1002/ana.21696 19557866

[hbm25121-bib-0022] Landau, S. M. , Fero, A. , Baker, S. L. , Koeppe, R. , Mintun, M. , Chen, K. , … Jagust, W. J. (2015). Measurement of longitudinal β‐amyloid change with 18F‐florbetapir PET and standardized uptake value ratios. Journal of Nuclear Medicine: Official Publication, Society of Nuclear Medicine, 56(4), 567–574. 10.2967/jnumed.114.148981 PMC5313473 25745095

[hbm25121-bib-0023] Landau, S. M. , Horng, A. , Jagust, W. J. , & Alzheimer's Disease Neuroimaging Initiative . (2018). Memory decline accompanies subthreshold amyloid accumulation. Neurology, 90(17), e1452–e1460. 10.1212/WNL.0000000000005354 29572282 PMC5921038

[hbm25121-bib-0024] Landau, S. M. , Thomas, B. A. , Thurfjell, L. , Schmidt, M. , Margolin, R. , Mintun, M. , … Alzheimer's Disease Neuroimaging Initiative . (2014). Amyloid PET imaging in Alzheimer's disease: A comparison of three radiotracers. European Journal of Nuclear Medicine and Molecular Imaging, 41(7), 1398–1407. 10.1007/s00259-014-2753-3 24647577 PMC4055504

[hbm25121-bib-0025] Marinescu, R. V. , Eshaghi, A. , Lorenzi, M. , Young, A. L. , Oxtoby, N. P. , Garbarino, S. , … Alexander, D. C. (2019). DIVE: A spatiotemporal progression model of brain pathology in neurodegenerative disorders. NeuroImage, 192, 166–177. 10.1016/j.neuroimage.2019.02.053 30844504

[hbm25121-bib-0026] Matsubara, K. , Ibaraki, M. , Shimada, H. , Ikoma, Y. , Suhara, T. , Kinoshita, T. , & Ito, H. (2016). Impact of spillover from white matter by partial volume effect on quantification of amyloid deposition with [11C]PiB PET. NeuroImage, 143, 316–324. 10.1016/j.neuroimage.2016.09.028 27639351

[hbm25121-bib-0027] Mattsson, N. , Insel, P. S. , Donohue, M. , Landau, S. , Jagust, W. J. , Shaw, L. M. , … Weiner, M. W. (2015). Independent information from cerebrospinal fluid amyloid‐β and florbetapir imaging in Alzheimer's disease. Brain, 138(Pt 3), 772–783. 10.1093/brain/awu367 25541191 PMC4339769

[hbm25121-bib-0028] Mattsson, N. , Palmqvist, S. , Stomrud, E. , Vogel, J. , & Hansson, O. (2019). Staging β‐amyloid pathology with amyloid positron emission tomography. JAMA Neurology, 76, 1319 10.1001/jamaneurol.2019.2214 PMC6646987 31314895

[hbm25121-bib-0029] Müller‐Gärtner, H. W. , Links, J. M. , Prince, J. L. , Bryan, R. N. , McVeigh, E. , Leal, J. P. , … Frost, J. J. (1992). Measurement of radiotracer concentration in brain gray matter using positron emission tomography: MRI‐based correction for partial volume effects. Journal of Cerebral Blood Flow and Metabolism: Official Journal of the International Society of Cerebral Blood Flow and Metabolism, 12(4), 571–583. 10.1038/jcbfm.1992.81 1618936

[hbm25121-bib-0030] Navitsky, M. , Joshi, A. D. , Kennedy, I. , Klunk, W. E. , Rowe, C. C. , Wong, D. F. , … Devous, M. D., Sr. (2018a). Standardization of amyloid quantitation with florbetapir standardized uptake value ratios to the Centiloid scale. Alzheimer's & Dementia: The Journal of the Alzheimer's Association, 14(12), 1565–1571. 10.1016/j.jalz.2018.06.1353 30006100

[hbm25121-bib-0032] Ni, R. , Gillberg, P.‐G. , Bergfors, A. , Marutle, A. , & Nordberg, A. (2013). Amyloid tracers detect multiple binding sites in Alzheimer's disease brain tissue. Brain: A Journal of Neurology, 136(Pt 7), 2217–2227. 10.1093/brain/awt142 23757761

[hbm25121-bib-0033] Ottoy, J. , Verhaeghe, J. , Niemantsverdriet, E. , Wyffels, L. , Somers, C. , De Roeck, E. , … Staelens, S. (2017). Validation of the Semiquantitative static SUVR method for 18F‐AV45 PET by pharmacokinetic Modeling with an arterial input function. Journal of Nuclear Medicine: Official Publication, Society of Nuclear Medicine, 58(9), 1483–1489. 10.2967/jnumed.116.184481 28336779

[hbm25121-bib-0034] Palmqvist, S. , Schöll, M. , Strandberg, O. , Mattsson, N. , Stomrud, E. , Zetterberg, H. , … Hansson, O. (2017). Earliest accumulation of β‐amyloid occurs within the default‐mode network and concurrently affects brain connectivity. Nature Communications, 8(1), 1214 10.1038/s41467-017-01150-x PMC5663717 29089479

[hbm25121-bib-0035] Reitan, R. M. (1958). Validity of the trail making test as an indicator of organic brain damage. Perceptual and Motor Skills, 8(3), 271–276. 10.2466/pms.1958.8.3.271

[hbm25121-bib-0036] Rodrigue, K. M. , Kennedy, K. M. , Devous, M. D., Sr. , Rieck, J. R. , Hebrank, A. C. , Diaz‐Arrastia, R. , … Park, D. C. (2012). β‐Amyloid burden in healthy aging: Regional distribution and cognitive consequences. Neurology, 78(6), 387–395. 10.1212/WNL.0b013e318245d295 22302550 PMC3280058

[hbm25121-bib-0037] Rullmann, M. , Dukart, J. , Hoffmann, K.‐T. , Luthardt, J. , Tiepolt, S. , Patt, M. , … Barthel, H. (2016). Partial‐volume effect correction improves quantitative analysis of 18F‐Florbetaben β‐amyloid PET scans. Journal of Nuclear Medicine: Official Publication, Society of Nuclear Medicine, 57(2), 198–203. 10.2967/jnumed.115.161893 26541776

[hbm25121-bib-0038] Sabri, O. , Seibyl, J. , Rowe, C. , & Barthel, H. (2015). Beta‐amyloid imaging with florbetaben. Clinical and Translational Imaging, 3(1), 13–26. 10.1007/s40336-015-0102-6 25741488 PMC4339690

[hbm25121-bib-0039] Sakr, F. A. , Grothe, M. J. , Cavedo, E. , Jelistratova, I. , Habert, M.‐O. , Dyrba, M. , … Alzheimer Precision Medicine Initiative (APMI) . (2019). Applicability of in vivo staging of regional amyloid burden in a cognitively normal cohort with subjective memory complaints: the INSIGHT‐preAD study. Alzheimer's Research & Therapy, 11(1), 15 10.1186/s13195-019-0466-3 PMC6357385 30704537

[hbm25121-bib-0040] Schmidt, M. E. , Chiao, P. , Klein, G. , Matthews, D. , Thurfjell, L. , Cole, P. E. , … Alzheimer's Disease Neuroimaging Initiative . (2015). The influence of biological and technical factors on quantitative analysis of amyloid PET: Points to consider and recommendations for controlling variability in longitudinal data. Alzheimer's & Dementia: The Journal of the Alzheimer's Association, 11(9), 1050–1068. 10.1016/j.jalz.2014.09.004 25457431

[hbm25121-bib-0041] Schoenberg, M. R. , Dawson, K. A. , Duff, K. , Patton, D. , Scott, J. G. , & Adams, R. L. (2006). Test performance and classification statistics for the Rey auditory verbal learning test in selected clinical samples. Archives of Clinical Neuropsychology: The Official Journal of the National Academy of Neuropsychologists, 21(7), 693–703. 10.1016/j.acn.2006.06.010 16987634

[hbm25121-bib-0042] Seo, S. W. , Ayakta, N. , Grinberg, L. T. , Villeneuve, S. , Lehmann, M. , Reed, B. , … Rabinovici, G. D. (2017). Regional correlations between [11C]PIB PET and post‐mortem burden of amyloid‐beta pathology in a diverse neuropathological cohort. NeuroImage. Clinical, 13, 130–137. 10.1016/j.nicl.2016.11.008 27981028 PMC5144753

[hbm25121-bib-0043] Shaw, L. M. , Vanderstichele, H. , Knapik‐Czajka, M. , Clark, C. M. , Aisen, P. S. , Petersen, R. C. , … Alzheimer's Disease Neuroimaging Initiative . (2009). Cerebrospinal fluid biomarker signature in Alzheimer's disease neuroimaging initiative subjects. Annals of Neurology, 65(4), 403–413. 10.1002/ana.21610 19296504 PMC2696350

[hbm25121-bib-0044] Sojkova, J. , Goh, J. , Bilgel, M. , Landman, B. , Yang, X. , Zhou, Y. , … Resnick, S. M. (2015). Voxelwise relationships between distribution volume ratio and cerebral blood flow: Implications for analysis of β‐amyloid images. Journal of Nuclear Medicine: Official Publication, Society of Nuclear Medicine, 56(7), 1042–1047. 10.2967/jnumed.114.151480 PMC5367770 25977462

[hbm25121-bib-0045] Sperling, R. , Mormino, E. , & Johnson, K. (2014). The evolution of preclinical Alzheimer's disease: Implications for prevention trials. Neuron, 84(3), 608–622. 10.1016/j.neuron.2014.10.038 25442939 PMC4285623

[hbm25121-bib-0046] Su, Y. , Blazey, T. M. , Owen, C. J. , Christensen, J. J. , Friedrichsen, K. , Joseph‐Mathurin, N. , … Dominantly Inherited Alzheimer Network . (2016). Quantitative amyloid imaging in autosomal dominant Alzheimer's disease: Results from the DIAN study group. PLoS One, 11(3), e0152082 10.1371/journal.pone.0152082 27010959 PMC4807073

[hbm25121-bib-0047] Teipel, S. J. , Dyrba, M. , Chiesa, P. A. , Sakr, F. , Jelistratova, I. , Lista, S. , … Grothe, M. J. (2020). In vivo staging of regional amyloid deposition predicts functional conversion in the preclinical and prodromal phases of Alzheimer's disease. Neurobiology of Aging, 93, 98–108. 10.1016/j.neurobiolaging.2020.03.011 32291113

[hbm25121-bib-0048] Thal, D. R. , Beach, T. G. , Zanette, M. , Lilja, J. , Heurling, K. , Chakrabarty, A. , … Smith, A. P. L. (2018). Estimation of amyloid distribution by [18F]flutemetamol PET predicts the neuropathological phase of amyloid β‐protein deposition. Acta Neuropathologica, 136(4), 557–567. 10.1007/s00401-018-1897-9 30123935 PMC6132944

[hbm25121-bib-0049] Thal, D. R. , Rüb, U. , Orantes, M. , & Braak, H. (2002). Phases of a beta‐deposition in the human brain and its relevance for the development of AD. Neurology, 58(12), 1791–1800. Retrieved from. https://www.ncbi.nlm.nih.gov/pubmed/12084879 12084879 10.1212/wnl.58.12.1791

[hbm25121-bib-0050] Villain, N. , Chételat, G. , Grassiot, B. , Bourgeat, P. , Jones, G. , Ellis, K. A. , … Villemagne, V. L. (2012). Regional dynamics of amyloid‐β deposition in healthy elderly, mild cognitive impairment and Alzheimer's disease: A voxelwise PiB‐PET longitudinal study. Brain, 135(Pt 7), 2126–2139. 10.1093/brain/aws125 22628162

[hbm25121-bib-0051] Villemagne, V. L. , Ong, K. , Mulligan, R. S. , Holl, G. , Pejoska, S. , Jones, G. , … Rowe, C. C. (2011). Amyloid imaging with (18)F‐florbetaben in Alzheimer disease and other dementias. Journal of Nuclear Medicine: Official Publication, Society of Nuclear Medicine, 52(8), 1210–1217. 10.2967/jnumed.111.089730 21764791

[hbm25121-bib-0052] Villeneuve, S. , Rabinovici, G. D. , Cohn‐Sheehy, B. I. , Madison, C. , Ayakta, N. , Ghosh, P. M. , … Jagust, W. (2015). Existing Pittsburgh compound‐B positron emission tomography thresholds are too high: Statistical and pathological evaluation. Brain: A Journal of Neurology, 138(Pt 7, 2020–2033. 10.1093/brain/awv112 25953778 PMC4806716

[hbm25121-bib-0053] Wang, H. , & Song, M. (2011). Ckmeans.1d.Dp: Optimal k‐means clustering in one dimension by dynamic programming. The R Journal, 3(2), 29–33. 27942416 PMC5148156

