# Supplementary material for: Longitudinal validity of PET‐based staging of regional amyloid deposition
Source: Hum Brain Mapp. 2020 Jul 10;41(15):4219–31. doi: 10.1002/hbm.25121 (PMC7502828; doi:10.1002/hbm.25121)
Supplement: Supplementary file 1 — Appendix S1. Supporting Information [file HBM-41-4219-s001.pdf]

SUPPLEMENTAL FIGURE 1.

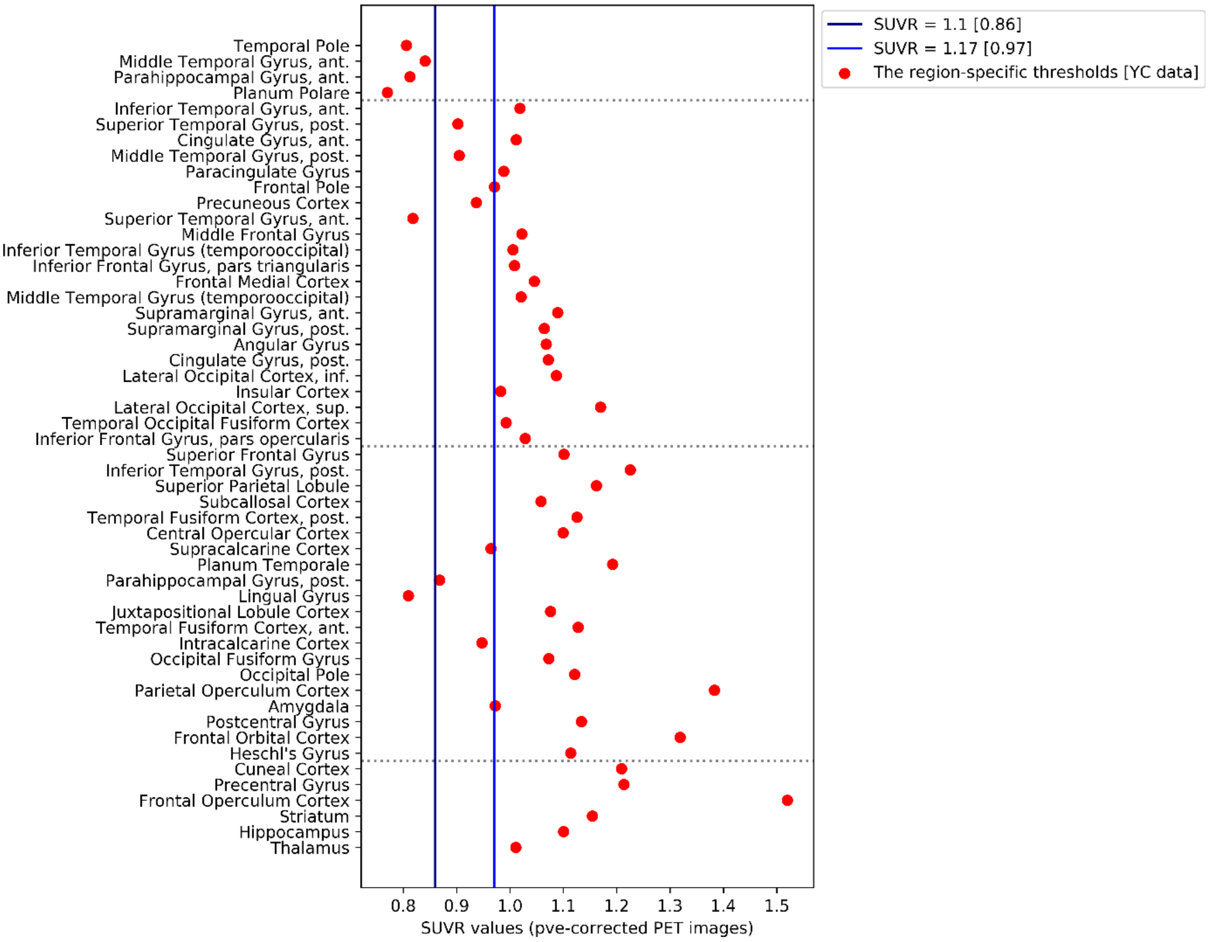

SUPPLEMENTAL FIGURE 1. The region-specific amyloid positivity thresholds (in pve-corrected data). Red scatterplot displays amyloid-positivity threshold for each of the regions. Dark blue lines display the commonly applied amyloid-positivity thresholds converted to the pve-corrected values (in square brackets). Brain regions are sorted according to the estimated progression model based on regional frequency values in CN data. Grey dotted lines indicate borders between anatomic divisions according to the 4-stage model.

SUPPLEMENTAL FIGURE 2

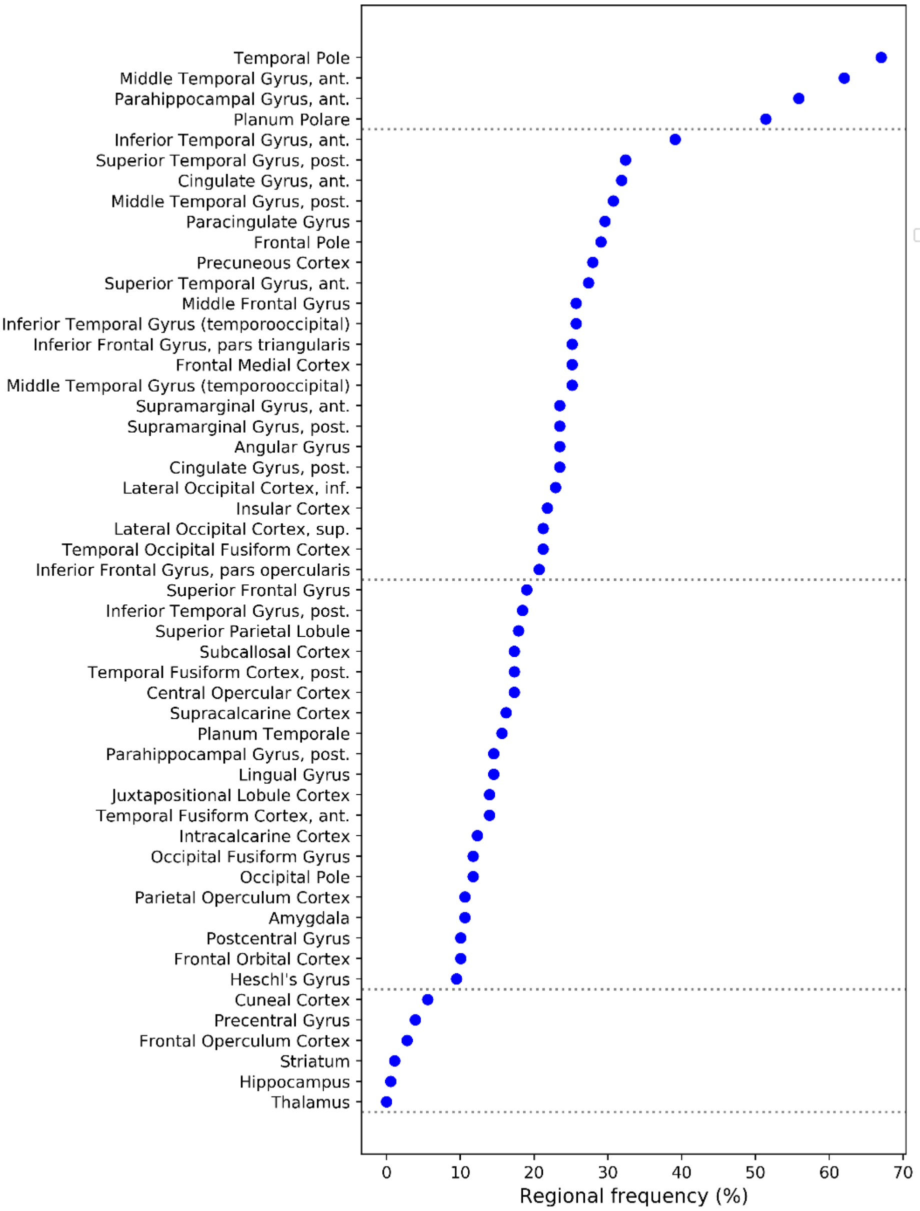

SUPPLEMENTAL FIGURE 2. The region-specific amyloid-positivity across 179 cognitively unimpaired older individuals. Blue scatterplot displays amyloid-positivity frequency for each of the regions. Brain regions are sorted by regional frequency values. Grey dotted lines indicate borders between anatomic divisions according to the 4-stage model.

SUPPLEMENTAL FIGURE 3

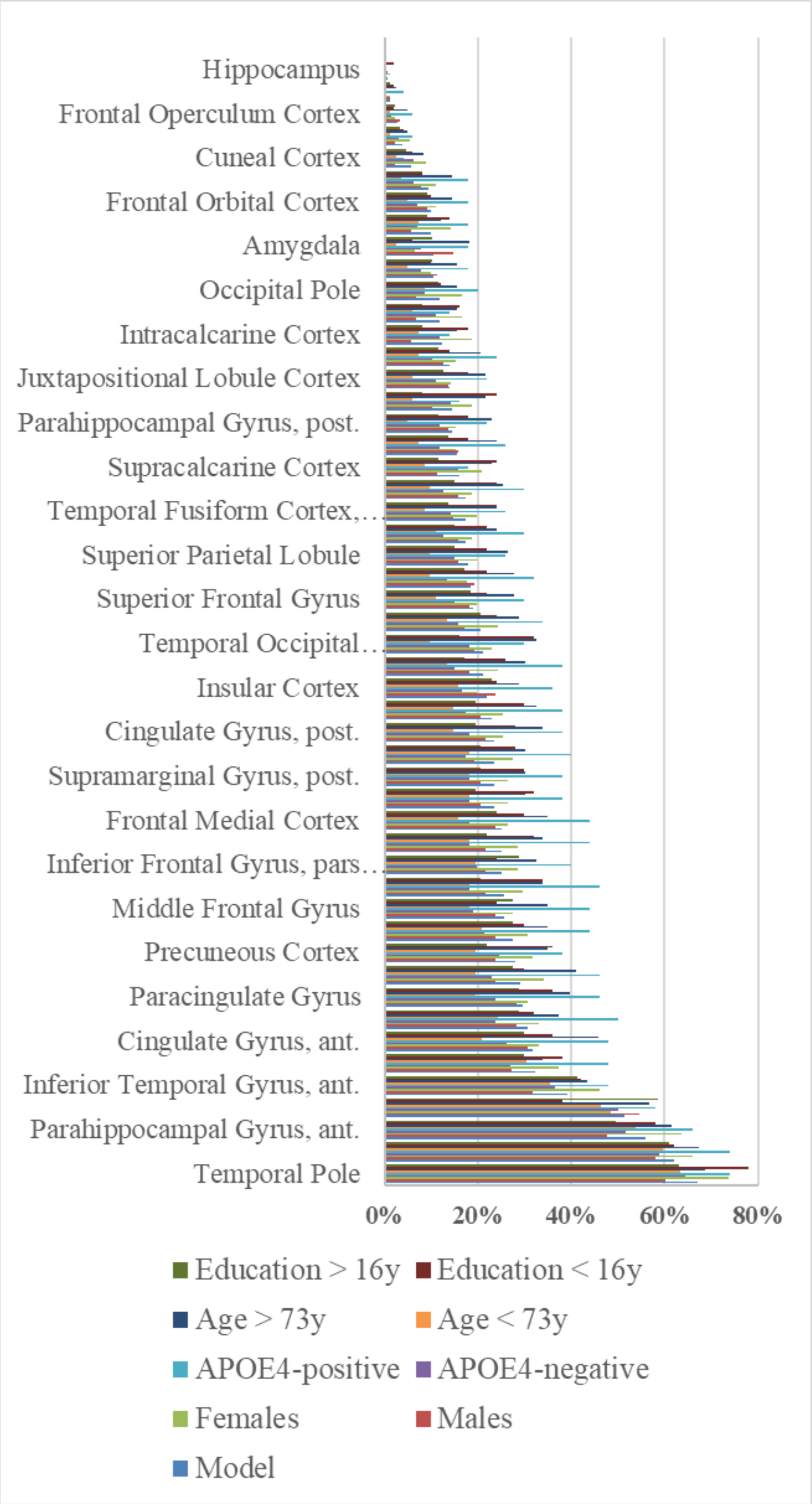

Supplemental Figure 3. Regional frequency models by subgroup. The figure displays regional frequencies in separate CN subgroups stratified by (1) males and females, (2) APOE4 carriers and non-carriers, (3) individuals older and younger than the group's median age (73y), (4) individuals with more or less than the group's median education years (16y). All models are plotted against the frequency model estimated from the whole group of 179 CN individuals.

SUPPLEMENTAL FIGURE 4

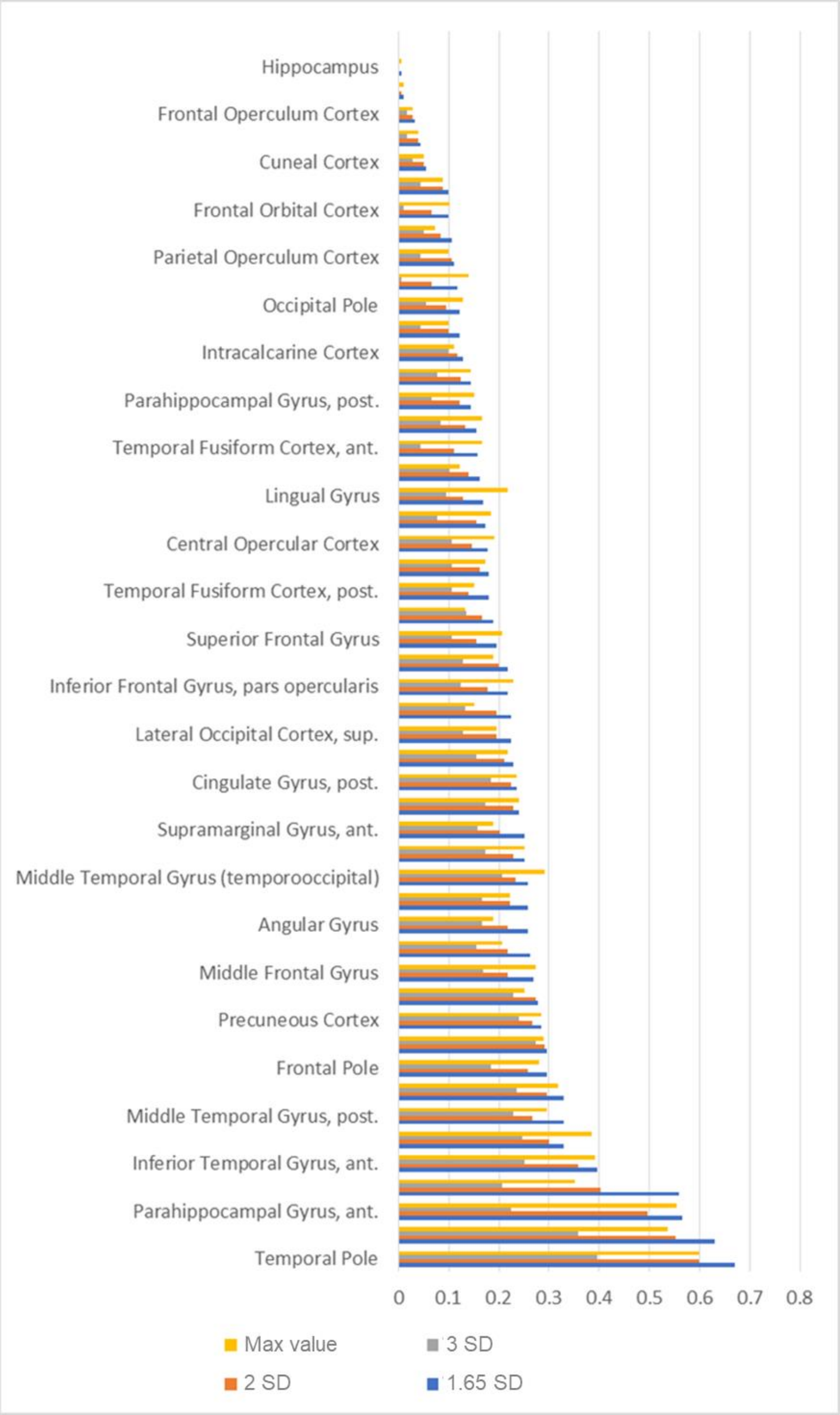

Supplemental Figure 4. Regional frequency models by different cut-off definitions. Frequency of regional amyloid-positivity across cognitively normal older individuals for regional cut-off definitions based on 1.65 (blue), 2 (orange), and 3 (grey) standard deviations above the mean value in the young control sample, as well as using the maximum value observed in this sample (yellow). Regions are ordered according to the principal model based on 1.65 standard deviations above the mean (blue).

SUPPLEMENTAL FIGURE 5

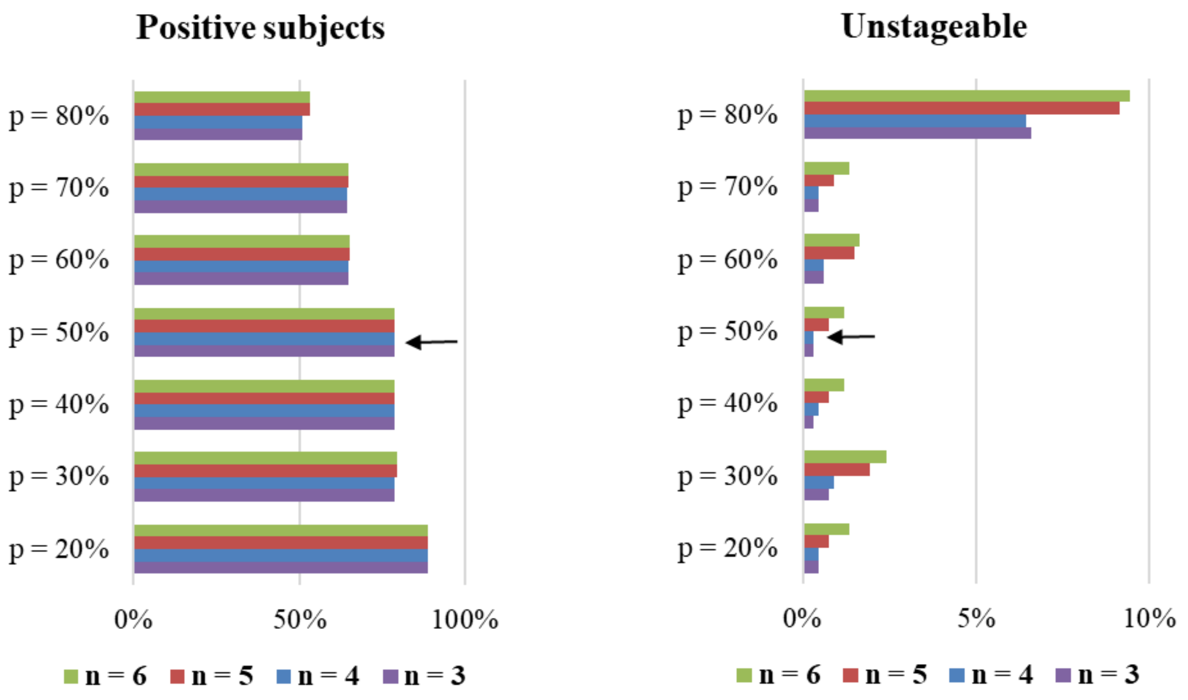

Supplemental Figure 5. Proportion of regionally positive (left) and unstageable (right) individuals by number of considered anatomical divisions in the staging model (n) and percentage (p) of regions within an anatomical division that must be positive for the division to be considered positive. The black arrows mark the selected values in the present study (i.e. n = 4 divisions and p = 50%) as adopted from the description of the original staging model (Grothe et al. 2017). The number of unstageable subjects generally increases for regionally more detailed staging models (n > 4), whereas it is similarly low for a less detailed model based on 3 anatomical divisions. As expected, the number of subjects with evidence of regional amyloid-positivity (positive in any division) increases for lower values of p. However, the number of unstageable cases remains either similarly low or increases for both lower and higher values of p compared to the initially chosen 50% limit. Thus, the a priori parameter settings (n=4, p=50%) appear to offer a good trade-off that allows sensitive detection of regional amyloid-positivity in a relatively detailed staging model while keeping the number of unstageable subjects as low as possible.

SUPPLEMENTAL FIGURE 6

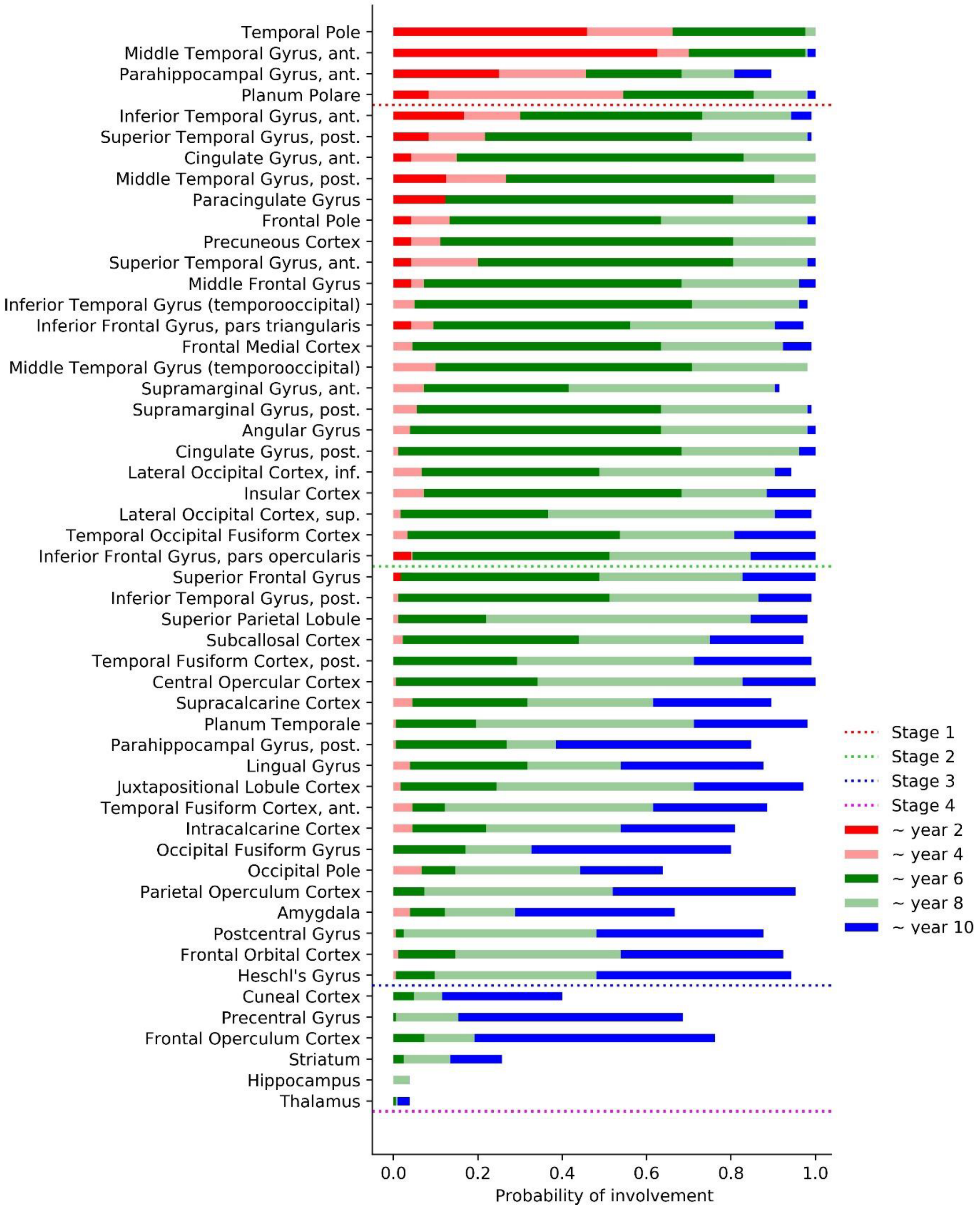

Supplemental Figure 6. Regional amyloid progression in the longitudinal probabilistic model. The diagram shows the estimated probability of a region to become amyloid-positive in consecutive 2-year intervals modelled from serial amyloid-PET acquisitions. The probability of a region to become involved at year 2 is based on the observed changes in subjects that were all-negative at baseline and is displayed in bright red colour. This data was used to select a subset of subjects that showed the corresponding 2-year changes at baseline, and their changes on follow-up amyloid-PET data were used to estimate the regional probabilities for a 4-year interval (faint red colour). Each consecutive 2-year interval was modelled in the same way (6th year displayed in bright green, 8th year in pale green, 10th year in blue). The assignment of progression year is an approximation that does not correspond to actual individual follow-up times. For direct comparison the regions are ordered according to their involvement in the cross-sectional progression model and dotted lines correspond to the respective progression stages.
